# Improved PCR-RFLP for the Detection of Diminazene Resistance in *Trypanosoma congolense* under Field Conditions Using Filter Papers for Sample Storage

**DOI:** 10.1371/journal.pntd.0001223

**Published:** 2011-07-26

**Authors:** Hervé Sèna Vitouley, Erick Ouma Mungube, Emmanuel Allegye-Cudjoe, Oumar Diall, Zakaria Bocoum, Boucader Diarra, Thomas F. Randolph, Burkhard Bauer, Peter-Henning Clausen, Dirk Geysen, Issa Sidibe, Zakaria Bengaly, Peter Van den Bossche, Vincent Delespaux

**Affiliations:** 1 Centre International de Recherche-Développement sur l'Elevage en zone Subhumide (CIRDES), Bobo Dioulasso, Burkina Faso; 2 Institute for Parasitology and Tropical Veterinary Medicine, Freie Universität Berlin, Berlin, Germany; 3 Central Veterinary Laboratories, Pong-Tamale, Tamale, Ghana; 4 International Livestock Research Institute (ILRI), Bamako, Mali; 5 Laboratoire Central Vétérinaire (LCV), Bamako, Mali; 6 Pan African Tsetse and Typanosomosis Eradication Programme (PATTEC), Bamako, Mali; 7 International Livestock Research Institute (ILRI), Nairobi, Kenya; 8 Institut of Tropical Medicine, Antwerp, Belgium; 9 Department of Veterinary Tropical Diseases, Faculty of Veterinary Science, University of Pretoria, Onderstepoort, South Africa; Yale School of Public Health, United States of America

Animal African trypanosomiasis (AAT) is caused by different species of the protozoan parasite *Trypanosoma* and affects a wide range of domestic animals. *Trypanosoma congolense* is widespread in the whole of sub-Saharan Africa and is the species causing considerable losses in livestock production, often affecting the health status of humans through endangering the food supply of rural communities. It is estimated that 50 million cattle are at risk of the disease and that the direct and indirect annual losses related to AAT reach US$4.5 billion [1].

Despite ambitious AAT control programmes such as the Pan African Tsetse and Trypanosomiasis Eradication Campaign that aim to eradicate the disease from the African continent, the control of the disease will in the foreseeable future continue to rely heavily on the use of trypanocidal drugs. Diminazene aceturate (DA) is the cheapest and most popular trypanocide. However, this drug, commercialized more than half a century ago, tends to become ineffective due to the spread of trypanocidal drug resistance now reported in 17 African countries [2].

The different techniques for the diagnosis of trypanocidal drug resistance were exhaustively reviewed by Delespaux et al. [2]. Two techniques are routinely used in the field namely, block treatments [3] and in vivo tests [4]. Block treatment involves follow-up for a period of 2 months of treated and non-treated (control) herds. This method provides information on the hazard of contracting trypanosomiasis (number of cattle becoming positive in the control group) and on the level of trypanocidal drug resistance (number of cattle becoming positive in the treated group). In vivo tests (performed in small ruminants for *Trypanosoma vivax* or in rodents for *T. congolense*) remain the gold standards but present some drawbacks, such as (i) problems associated with the growth of some *T. congolense* strains in rodents, (ii) the long period of follow up (2 months), and (iii) the intensive use of experimental animals.

To address these important drawbacks, the Department of Animal Health of the Institute of Tropical Medicine Antwerp has been actively involved in research on trypanocidal drug resistance and in 2007 was designated Reference Centre for Livestock Trypanosomiasis, Parasite Management and Diagnosis (RCLT) by the Food and Agriculture Organization of the United Nations (FAO) and accredited to ISONORM 17025 in 2009. Here, a *Bcl*I-PCR-RFLP was developed for the molecular diagnosis of DA resistance in *T. congolense* [5], the most widely used trypanocidal drug. The test is based on the detection of a single point mutation in P2-type purine transporters and has been transferred to a network of regional reference laboratories in West, East, and Southern Africa for use in large-scale surveys to detect the presence of trypanocidal drug resistance throughout tsetse-infested Africa. The correlation of this PCR-RFLP with the single dose mouse test (gold standard) [4] is excellent even if the PCR-RFLP was found to be more sensitive than the single dose mouse test [5], [6]. Although the test performs very well under laboratory conditions [6], it requires further adaptation and evaluation for use under field conditions. More specifically, the test's ability to amplify low concentrations of parasite DNA (as a result of often low parasitaemias in livestock) needs to be enhanced and its specificity improved by preventing incomplete digestion of the amplicon showing the point mutation by the *Bcl*I enzyme. This incomplete digestion creates profiles consisting of partially undigested and digested amplicons (resistant) and as such, falsely mixed RFLP profiles. Finally, sample collection, storage, and transfer to regional reference laboratories need to be simplified.

**Figure 1:**
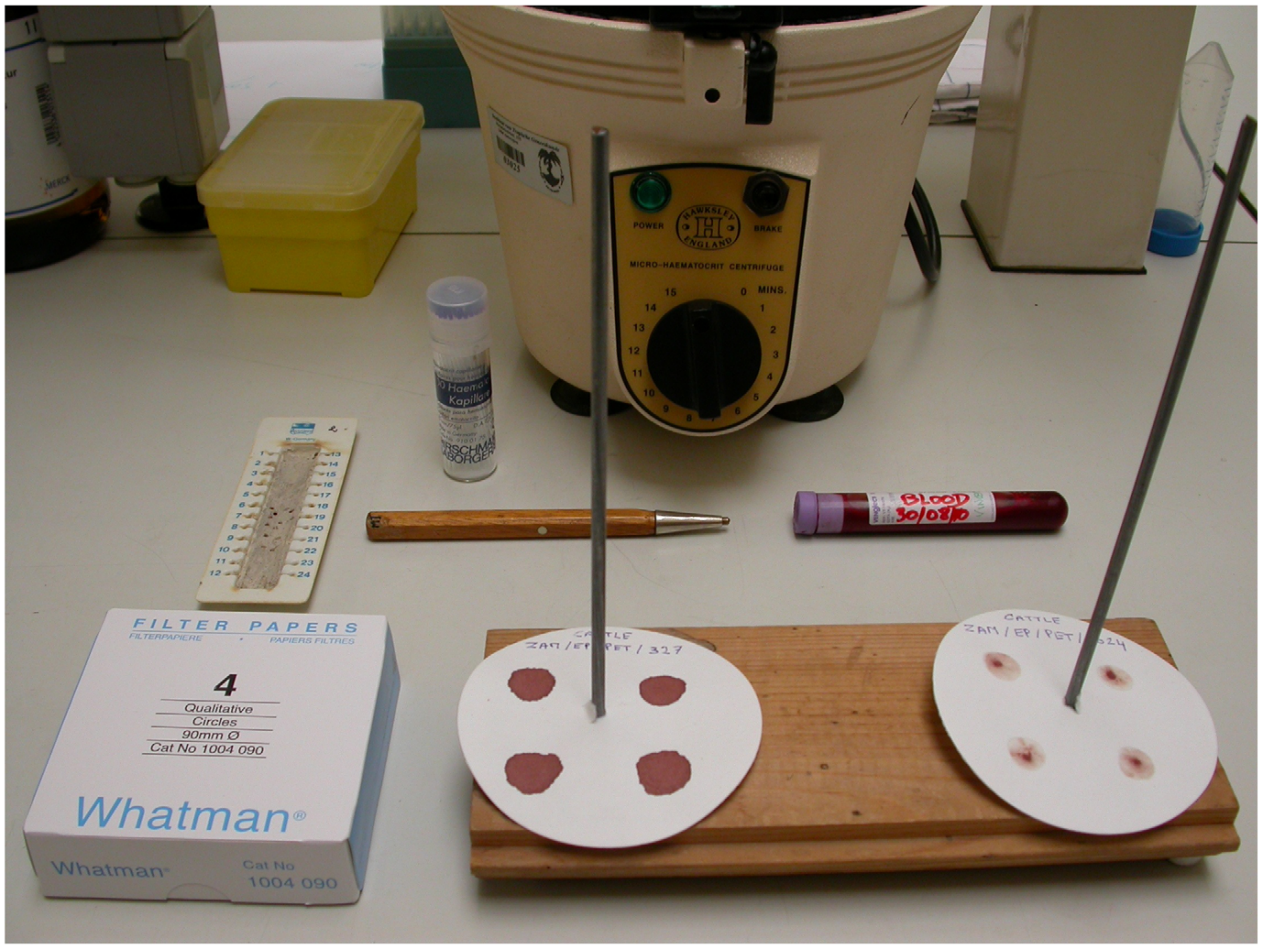
Laboratory material used for the processing of the samples.

**Figure 2:**
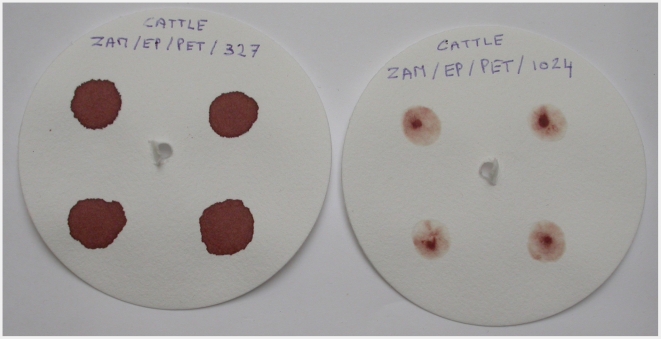
Dried blood spots (left) and buffy coats (right) on labelled filter papers.

In view of the above, the test's ability to amplify low concentrations of DNA was enhanced by adding a step of whole genome amplification using the QIAGEN REPLI-g UltraFast Mini Kit. Its specificity was improved by replacing the *Bcl*I (T^∧^GATCA) enzyme by *Dpn*II (^∧^GATC). Internal negative and positive controls (resistant and sensitive strains characterized by sequencing their respective amplicons) were added to ensure the absence of contamination, an effective DNA amplification, and the complete digestion of the PCR products. Finally, sample collection, storage, and transfer could be facilitated by collecting blood spots or buffy coats on filter paper (Whatman N°4, Whatman) (Figures 1 and 2). When costs and availability are not a limitation, the use of Whatman FTA or FTA Elute cards could constitute an interesting alternative, as it is believed to offer superior preservation of the DNA.

To evaluate the performance of the improved PCR-RFLP using the *Dpn*II enzyme for the detection of DA resistance in *T. congolense*, use was made of 449 whole blood spots (40–50 µl) on filter papers collected from parasitologically positive cattle originating from the cotton zone of Southern Mali where significant resistance to trypanocidal drugs was reported [7]. The samples were stored sun-protected for 1 year at tropical ambient temperature without adding dehydrated silica crystals in the storage plastic bags (i.e., stored under suboptimal conditions as often is the case in rural environments), allowing degradation of the DNA.

A total of 68% (304) of all blood spots was found positive for the presence of trypanosomes using the 18S pan-PCR developed by Geysen et al. [8]. Of these, 74% (225) were diagnosed as a single or mixed *T. congolense* infection.

Out of these 225 *T. congolense*–positive samples, 26% (59) amplified using the *DPn*II-PCR-RFLP. By adding a step of whole genome amplification, an extra 42 samples became positive, reaching a total of 44.9% (101).

**Figure 3:**
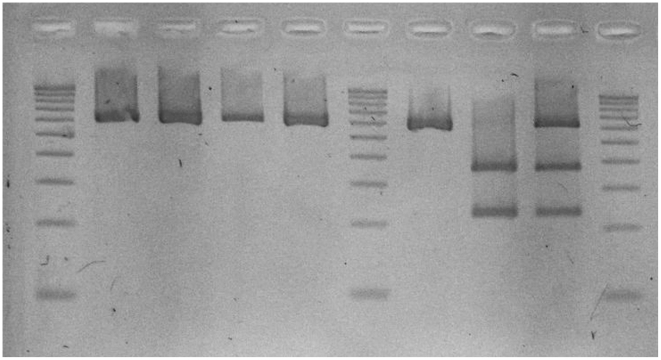
Output of the *DPn*II-PCR-RFLP with lanes 1, 6, and 10 as size markers (100-bp ladder), lanes 2, 3, 4, 5, and 7 as sensitive profiles (one band), lane 8 as resistant profile (two bands), and lane 9 as mixed profile (three bands).

The *DPn*II-PCR-RFLP profiles of the 101 amplified *T. congolense* samples were distributed as follows: 92% were resistant, 2% sensitive, and 6% presented a mixed profile (Figure 3). Even if due to host-related factors not all animals with those infections would fail to respond to treatment, such a high prevalence of DA resistance is more than worrying. As proposed by Clausen et al. [7], in such circumstances an integrated approach should be used based on (i) rational drug use information given to farmers, (ii) training farmers and paravets in integrated trypanosomiasis control, and (iii) training animal care providers to a more specific diagnosis of trypanosomiasis to avoid treatments inappropriate for the clinical signs.

Notwithstanding the suboptimal storage procedure, it can be concluded that blood samples collected and stored on filter papers can be used for detecting the presence of trypanosomes resistant to DA in a trypanosome population. Even under suboptimal conditions, 44.9% of the *T. congolense* samples were successfully characterized for DA resistance sufficient to estimate the prevalence and repartition of DA resistance in the field. Moreover, losses due to suboptimal storage conditions can be compensated for by increasing the sample size. Hence, considering the important logistical and economic advantages of filter papers, their use greatly facilitates the implementation of large-scale surveys for trypanocidal drug resistance in trypanosomiasis-affected African countries using molecular diagnostic tools. Moreover, their use greatly supports the functioning and sustainability of much-needed regional reference laboratories.

**Box 1.** Advantages and Disadvantages of using the *Dpn*II-PCR-RFLP for the Diagnosis of DA Resistance in *T. congolense* on Blood Samples Collected on Filter Papers

**Advantages**

1. Easy collection/storage/transfer of samples (blood spots or buffy coats on filter paper stored at ambient room temperature).
2. Allows even for the screening of large numbers of trypanosome strains circulating in remote areas without use of experimental animals.
3. Contributes to the sustainability of regional reference laboratories for trypanocidal drug resistance.

**Disadvantages**

1. Incorrect storage of filter papers reduces the number of PCR-positive samples.
2. The use of filter papers increases the danger of sample contamination.
3. Relatively high cost (25€ for species identification and DA resistance detection, all included except technician salary and transport) but still acceptable compared to a test in mice (150€ all included except technician salary and transport), tests in cattle or block treatment being even more expensive (e.g., €945 for a survey of treatment failure in a village herd).
